# Development of an Innovative Lightweight Composite Material with Thermal Insulation Properties Based on Cardoon and Polyurethane

**DOI:** 10.3390/polym16010137

**Published:** 2023-12-31

**Authors:** Raquel A. Fernandes, Nuno Ferreira, Sandro Lopes, Jorge Santos, Nelson Bento Pereira, Nuno Oliveira Ferreira, Lina Nunes, Jorge M. Martins, Luisa H. Carvalho

**Affiliations:** 1 ARCP Colab—Rede de Competências em Polímeros, Rua Júlio de Matos, 828/882, 4200-355 Porto, Portugal; raquel.fernandes@arcp.pt (R.A.F.); nuno.ferreira@arcp.pt (N.F.); sandro.lopes@arcp.pt (S.L.); jorge.ucha@arcp.pt (J.S.); 2 LEPABE—Faculty of Engineering, University of Porto, Rua Dr. Roberto Frias, s/n, 4200-465 Porto, Portugal; jmmartins@estgv.ipv.pt; 3 AliCE—Associate Laboratory in Chemical Engineering, Faculty of Engineering, University of Porto, Rua Dr. Roberto Frias, 4200-465 Porto, Portugal; 4 CICon—Center for Innovation in Construction, Zona Industrial de Sabroso de Aguiar-Lote 2B, 5450-371 Vila Pouca de Aguiar, Portugal; nelson@houselab.pt; 5 CEPAT—Center for Heritage Studies, Avenida do Conde 5643, 4465-097 São Mamede de Infesta, Portugal; nuno.ferreira@houselab.pt; 6 Secundino Queirós Construction, Avenida Lopes de Oliveira 29, 5450-140 Pedras Salgadas, Portugal; 7 LNEC—Laboratório Nacional de Engenharia Civil, Structures Department, Av. do Brasil, 101, 1700-066 Lisbon, Portugal; linanunes@lnec.pt; 8 DEMad—Department of Wood Engineering, Instituto Politécnico de Viseu, Campus Politécnico de Repeses, 3504-510 Viseu, Portugal

**Keywords:** cardoon, insulation, polyurethane, sustainable, composite, construction

## Abstract

The search for innovative and sustainable solutions to improve the energy efficiency of the construction industry has been a hot topic for researchers due to the tremendous impact of insulator materials in the thermal comfort of buildings. In the present work, an innovative lightweight composite material with thermal insulation properties was developed, for the first time, by using cardoon particles and polyurethane. The formulation of the composite material was optimized in terms of cardoon fraction and the polyol/isocyanate ratio, to achieve the best compromise between internal bond (IB) strength and thickness swelling (TS). The best performing composite was PU75-CP45, with 45 wt% of cardoon particles and 75% of isocyanate, achieving an IB of 0.41 MPa and a TS of 5.3%. Regarding insulation properties, the PU75-CP45 composite material exhibits a promising performance when compared to conventional construction industry materials by tuning its thickness. Additionally, the composite material presented very low emissions of volatile organic compounds and formaldehyde (bellow to legislation levels) and high resistance to biological degradation.

## 1. Introduction

The construction industry is one of the greatest contributors towards environmental issues, representing almost 50% of global carbon emissions, energy and natural resources’ consumption and solid waste production [1,2]. Nevertheless, the problems associated with this sector are not just limited to the production phase, as the life cycle of their final products has also a negative impact on environmental sustainability. According to the European Commission, buildings are responsible for more than 40% of global energy consumption and 36% of greenhouse gas emissions [3,4]. In fact, the largest fraction of the energetic demand of buildings is due to thermal comfort, leading thermal insulation to be a hot topic for the development of alternative and sustainable materials for construction industry [1,2,3,4].

Thermal insulation reduces the heat transfer across any surface, allowing the maintenance of a desired temperature, reducing the extra cooling or heating and, consequently, decreasing the energetic footprint of buildings [5]. The available thermal insulation materials are essentially classified into four different groups, namely inorganic, organic, combined and advanced materials [3,6]. In the construction industry, inorganic (glass wool and rock wool) and polymer-derived solutions (polyurethane, polyisocyanurate and polystyrene) are preferred in most cases due to their low price and thermal conductivity [3,6]. However, their use can cause some health problems, especially skin and lung irritation [3,7]. In this way, organic insulation materials, derived from natural resources, have become more attractive regarding their use in buildings construction [3,8]. Properties such as thermal conductivity, density, availability and price are determinant factors of the efficient performance of these innovative alternatives when compared to other commercial solutions [2].

Natural resources such as cellulose, straw, hay, linen and hemp have been applied for decades in the envelope of buildings to promote thermal insulation [9,10]. In recent years, the use of cellulose-based fibers in the fabrication of organic composite materials with insulating properties has been exploited [11,12,13]. Bellel et al. (2023) have proved the successful use of date palm fibers as a reinforcement material to improve the insulating properties of Portland cement [11]. Also, Belhous et al. (2023) revealed the potential of date palm fibers to regulate the indoor temperature of houses, with an increase of up to 3 °C in winter and a decrease of 5 °C in summer, by comparing it with non-insulating construction [12]. Bakatovich et al. (2019) developed an insulating composite material using moss as fiber, with low thermal conductivity and good mechanical properties [13]. Hussain et al. (2019) used hemp shiv in a lightweight insulator, with high compressive strain [14]. Corn stalks were also applied in the production of insulating composites by Ahmad et al. (2019) together with magnesium–phosphate cement [15]. Although natural fibers have gained interest as reinforcements for insulation purposes, their moisture absorption, poor wettability and, particularly, poor mechanical resistance have limited their application [7]. Therefore, cardoon (*Cynara cardunculus* L.) gains special attention due to its great mechanical properties [16].

Cardoon is a plant well established in the Mediterranean basin, well adapted to the climatic conditions [17,18]. In Portugal, cardoon has particular interest in the production of cheese due to the high content of proteases and coagulant factors present in its flowers [17,18]. However, the branches, stalks and lees are considered by-products and they are mainly used for producing energy and oils for human and animal consumption [19]. Besides its renewable and sustainable nature, this type of material has a lower density (between 70 and 220 kg m^−3^), making it an excellent option for the production of lightweight composite materials [20,21].

The present work aims to develop a lightweight composite material with insulation properties, using cardoon by-products and polyurethane (PU) adhesive. To the best of our knowledge, this is the first time that cardoon by-products are applied in the production of composite materials with insulation potential. The composite material combines insulation capacity with good physical–mechanical properties and non-hazardous manipulation, opening up a new route for the building sector. The impact of cardoon fraction on the mechanical properties of the produced materials is assessed, as well as the effect of PU formulation. The developed material should have a density ≤ 300 kg m^−3^ and suitable mechanical properties (internal bond strength ≥ 0.30 MPa), according to the standard requirements for general purpose lightweight particleboards for use in dry conditions (Type LP1)-(CEN/TS 16368) [22]. Its resistance in humid conditions is also tested, as well as insulation properties. A comparison is made with the conventional materials usually applied in the construction industry. In order to assess its contribution to the quality of indoor air, additional tests of formaldehyde and volatile organic compounds (VOCs) emissions are also performed.

## 2. Materials and Methods

### 2.1. Materials

Cardoon branches, stalks and leaves were supplied by Quinta da Cruz (Viseu, Portugal). The by-products were air-dried at (23 ± 1) °C and (50 ± 3)% of relative humidity until reaching the equilibrium moisture (about 10%), ground in a cutting mill (Retsch SM300, Haan Germany) and sieved in a sieve shaker (Retsch AS300 Control, Haan Germany). The fraction of cardoon particles (CP) with 1 to 4 mm of mesh was selected. Polyol (Daltoflex EC21060) with a hydroxyl value of 278.8 mg KOH g^−1^ and density of 1.03 g cm^−3^, and methylene diphenyl diisocianate (MDI, Suprasec 6506) with an NCO content of 29.3% was purchased from Huntsman Corporation (Conroe, TX, USA).

### 2.2. Preparation of Polyurethane–Cardoon (PU-CP) Composite Panels

The PU-CP composite panels were produced by mixing a certain amount of cardoon particles (from 25 to 65 wt% of the total mass of composite) with the components of polyurethane resin (polyol and MDI), to reach the target density of 300 kg m^−3^. The impact of the polyol/MDI ratio was tested by varying the MDI content from 50 to 125 wt% regarding the mass of polyol. The production process was carried out under room temperature, and it was initiated by the mixing of polyol with the cardoon particles, using a mechanical stirrer until a homogeneous mixture was obtained. After that, MDI was added to the system, and stirring was maintained for an additional 45 s. In a second phase, the homogeneous mixture of cardoon particles and PU resin was transferred into a mold, with dimensions of 500 × 500 × 100 mm, and it was closed by keeping a constant pressure to avoid opening. The composite was left to cure for 30 min at room temperature and it was demolded after that period. After curing, the panels were dried at (20 ± 2) °C and relative humidity of (65 ± 5)% until a constant mass was obtained. The obtained materials were denoted PUx-CPy, where x refers to the MDI content (x = 50, 75, 100 and 125) and y refers to the content of cardoon particles in the composite (y = 25, 35, 45, 55 and 65). Table 1 presents the formulation of all materials produced by varying the cardoon and MDI contents.

A neat PU foam was produced by mixing a certain amount of polyol and MDI in a molar ratio of 1:1 under mechanical stirring at room temperature for 10 min. After this period, the foam was completely cured and ready to be tested.

### 2.3. Characterization Techniques

#### 2.3.1. Scanning Electron Microscopy (SEM)

The morphological characterization of the PU-CP composite panels was performed through scanning electron microscopy (SEM), using a high-resolution (Schottky) Environmental Scanning Electron Microscope with X-ray Microanalysis and Electron Backscattered Diffraction analysis: Quanta 400 FEG ESEM/EDAX Genesis X4M (FEI, Hillsboro, OR, USA). In order to improve the quality of analysis, the samples were coated with an Au/Pd thin film, via sputtering, using the SPI Module Sputter Coater equipment.

#### 2.3.2. Fourier-Transformed Infrared (FTIR) Spectroscopy

FTIR spectra were recorded on a VERTEX 70 FTIR spectrometer (BRUKER, Billerica, MA, USA) in transmittance mode and equipped with a high-sensitivity DLaTGS detector at room temperature. The samples were measured in ATR mode with no pretreatment, using an A225/Q PLATINUM ATR diamond crystal with a single reflection accessory. The spectra were recorded from 4000 to 400 cm^−1^ with a resolution of 4 cm^−1^. All spectra were recorded and processed with OPUS 7.0 software.

#### 2.3.3. Thermogravimetric Analysis (TGA)

TGA analysis was performed using a Netzsch STA 449 F3 Jupiter thermal instrument. The samples were heated up from 24 °C to 500 °C at 10 °C min^−1^ rate under air flow.

#### 2.3.4. Physical–Mechanical Evaluation

The physical–mechanical performance of composite panels was assessed according to the European standards for wood-based panels. For density (D) determination (EN 323:1993; [23]), 50 mm × 40 mm specimens were precisely measured (length and thickness) and weighed to determine the mass and volume.

To determine the moisture content (MC; EN 322:1993, [24]), the specimens were weighed before and after drying in an oven at (103 ± 2) °C until a constant mass was obtained. 

The internal bond strength (IB) was performed according EN 319:1993 [25]. For this, the square specimens of 40 mm of length were glued to a metal support and subjected to a tensile force until rupture. The load was applied at a constant rate (1 mm min^−1^) so that the maximum load was reached within (60 ± 3) s.

The determination of thickness swelling (TS) followed the standard procedure EN 317:1993 [26]. For that, the thickness of the square specimens of 40 mm of thickness was measured before and after immersion in water at (20 ± 3) °C for 24 h.

Every determination was carried out five times and the results were averaged.

#### 2.3.5. Moisture Resistance Tests

Dimensional stability was assessed according to EN 318:2002 [27]. For the analysis, the length and thickness of the 300 mm × 50 mm specimens were measured after storage under (20 ± 2) °C and different conditions of relative humidity (RH; 30, 65 and 85%) at a constant mass.

Moisture resistance was evaluated through cycle tests according to EN 321:2010 [28]. Briefly, 50 mm × 50 mm samples (length and thickness, respectively) were subjected to three consecutive cycles, with four stages each: (i) immersion in water at (20 ± 1) °C for (70 ± 1) h; (ii) freezing at −12 to −25 °C for (24 ± 1) h; (iii) drying in an oven at (70 ± 2) °C for (70 ± 1) h; and (iv) air cooling at (20 ± 5) °C for (4 ± 0.5) h. At the end of the test, the samples were subjected to IB determination (EN 319:1993, [25]) and TS was also measured (EN 317:1993, [26]). Additionally, the samples were also tested in boiling conditions according to EN 1087-1: 1995 [29]. In this method, 50 mm × 50 mm glued specimens were heated up to100 °C over (90 ± 10) min. After this period, boiling was maintained for (120 ± 5) min. After that, the samples were cooled through immersion in water at (20 ± 1) °C for 1–2 h and then tested for IB determination (EN 319:1993, [25]).

#### 2.3.6. Thermal Conductivity Measurements

Thermal conductivity was assessed through EN 12667:2001 [30]. For that, 300 × 300 × 40 mm^3^ PUx-CPy panels were firstly conditioned at 25 °C and 65% of relative humidity before being analyzed. After that, the sample was placed in a sealed chamber of the equipment Netzsch TCA 300 (Taurus Instruments, Fridingen, Germany) and the thermal conductivity was measured at 10 °C, 20 °C and 30 °C. The analysis was carried out in five different specimens of each composite panel and the results were averaged.

#### 2.3.7. Formaldehyde and Volatile Organic Compounds (VOCs) Emissions

The determination of formaldehyde emissions was carried out using the gas analysis method (EN ISO 12460-3: 2023, [31]). For this, 400 mm × 50 mm samples (with 20 mm of thickness) were externally sealed with aluminum foil and put in a closed chamber at (60 ± 0.5) °C, with a relative humidity below 3%, an air flow of (60 ± 3) L h^−1^ and a pressure of 1000–1200 Pa. The formaldehyde emission was measured after 4 h of test. The formaldehyde concentration was expressed in mg CH_2_O m^−2^ h^−1^.

The determination of VOCs was performed according to the standard test ISO 16000-9:2006 [32]. The samples were placed in a closed chamber at (23 ± 2) °C and (50 ± 5)% of relative humidity, under air flow (0.1 to 0.3 m s^−1^). The pressure inside the chamber was kept at 1000 Pa and the test was carried out for 28 days. The VOCs’ concentration was expressed in mg VOCs m^−2^ h^−1^. Both tests were carried out in duplicate.

#### 2.3.8. Susceptibility to Biological Degradation

The samples (approx. 50 mm × 25 mm × 15 mm thickness) were used to test the susceptibility of the composite material to subterranean termites (*Reticulitermes grassei*) according to an adaptation of EN 117:2012 [33] with smaller test specimens and 150 termite workers per flask.

The susceptibility to white rot fungus (*Pycnoporus sanguineus*) was assessed through the “mini-block method” [34].

Beech (*Fagus sylvatica* L.) untreated solid wood samples with similar dimensions were included as virulence controls.

## 3. Results and Discussion

### 3.1. Characterization of Raw Materials and PUx-CPy Composite Panels

**Figure 1:**
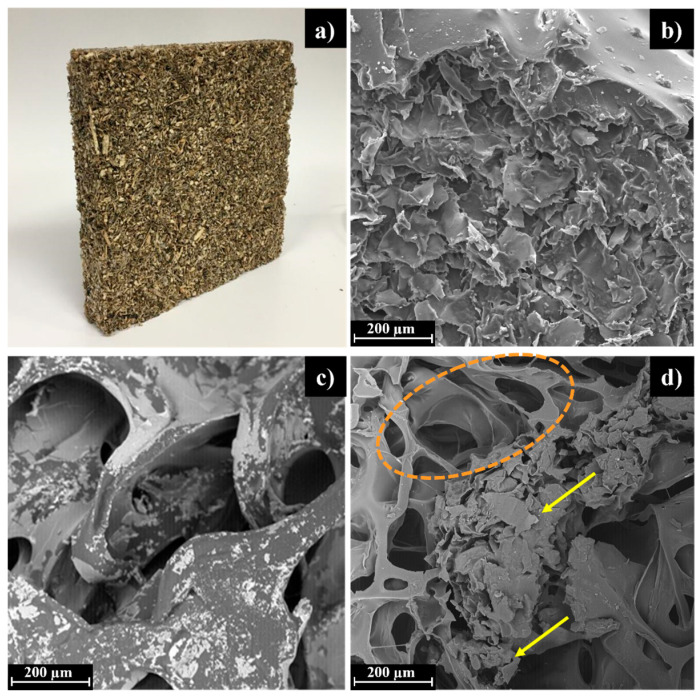
(**a**) Photograph of the PUx-CPy composite panel. SEM micrographs of (**b**) cardoon particles (CP), (**c**) PU foam and (**d**) PUx-CPy composite panel. Dashed orange line and yellow arrows indicate regions of PU foam and CP aggregates, respectively.

The morphology of PU foam, cardoon particles and an example of PUx-CPy was assessed through SEM analysis and the micrographs are presented in Figure 1.

The surface of PUx-CPy composite panels (Figure 1a) evidences the high variability of the particle size of cardoon (from 1 to 4 mm). At the microscopic level, cardoon particles (Figure 1b) present an irregular surface, with no defined structure, caused by the destruction during the milling process. Contrariwise, PU foam (Figure 1c) exhibits a well-defined honeycomb arrangement due to the release of carbon dioxide formed through the polymerization reactions between polyol, MDI and water [35,36,37,38,39]. Regarding these distinct morphologies, in the micrographs of the PUx-CPy panel (Figure 1d), it is possible to identify regions of CP (yellow arrows, Figure 1d) dispersed in the PU matrix (dashed orange line, Figure 1d), with significant interfacial interaction between the two components preventing the release of CP.

**Figure 2:**
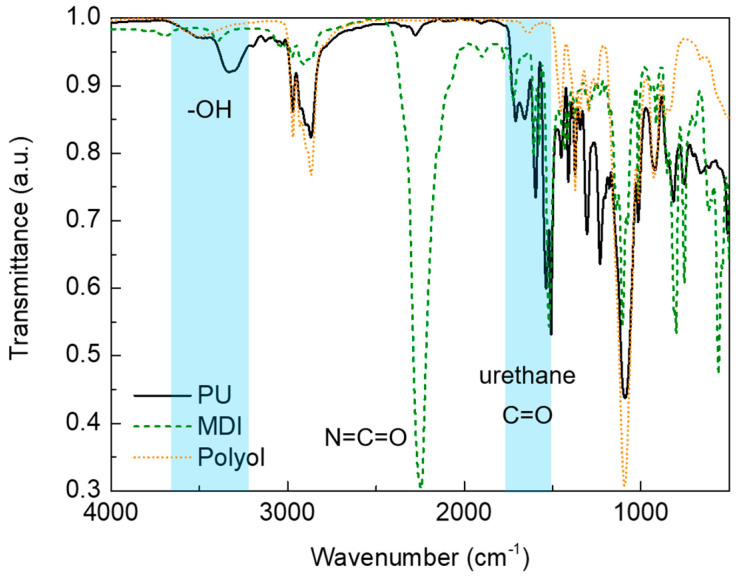
FTIR spectra of polyol (doted orange line), MDI (dashed green line) and PU (solid black line) foam. Blue zones and dashed blue line indicate specific FTIR bands.

**Figure 3:**
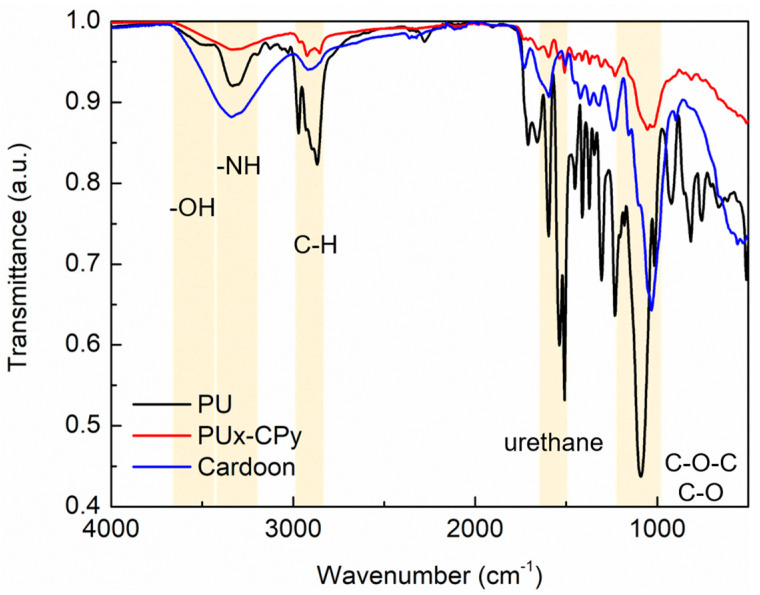
FTIR spectra of PU foam (black line), cardoon particles (blue line) and PUx-CPy composite material (red line). Yellow zones correspond to characteristic bands of transmittance.

FTIR analysis also corroborates the mechanism involved in the formation of PU foam (Figure 2) and the existence of chemical interactions between PU and cardoon particles (Figure 3).

According to the chemistry of PU foam, it is known that its formation may occur through a chemical reaction between hydroxyl and isocyanate groups [36,38,39,40]. FTIR analysis (Figure 2) corroborates the presence of these specific functional groups with a broad band at 3000–3500 cm^−1^ in polyol spectrum (-OH bond) and at 2273 cm^−1^ in the case of MDI spectrum (N=C=O group) [40,41,42,43,44]. Additionally, in the spectrum of MDI, an important band is also observed at 2094 cm^−1^, which is attributed to C-H stretching (Figure 2) [41,42,43,44,45].

Regarding the PU foam, the presence of -OH stretching vibrations from the unreacted polyol (3000 to 3500 cm^−1^; Figure 2) is observed [37,43,46]. In addition, the bands at 1231 cm^−1^ and 1090 cm^−1^ are assigned to the O-CO and C-O bonds of ethers (residues of industrial polyols) [26,32,35]. At 1709 cm^−1^ and 1509 cm^−1^, a characteristic band appears related to vibrations of urethane bond and the respective carbonyl group [37,43,46]. The band attributed to the isocyanate group (at 2273 cm^−1^; Figure 2) is negligible, indicating an almost complete consumption of this group in the formation of PU foam [37,43,46].

Cardoon particles belong to the group of lignocellulosic materials, which have a complex structure composed of cellulose, hemicellulose and lignin. By analyzing the FTIR spectrum (Figure 3), it is possible to identify some characteristic bands: 3000 to 3500 cm^−1^ is ascribed to O-H stretching vibrations of cellulose units, 2900 to 2800 cm^−1^ is related to the C-H bond in cellulose polymers, a peak at 1595 cm^−1^ is attributed to the hydrogen bridge between the hydroxyl groups and oxygen atoms of glucose monomers, at 1239 cm^−1^ there is a peak related to the C-O-C group of lignins, and at 1031 cm^−1^ there is a band revealing the existence of C-O groups from cellulosic fraction [37,43,46].

Regarding the PU-CP composite material, it is possible to confirm the presence of both cardoon and PU fraction due to the peaks ascribed to cellulosic units (2867 cm^−1^; Figure 3) and to the urethane group (1509 cm^−1^; Figure 3), respectively [37,43,46]. Additionally, the stretching vibrations of C-O-C and C-O groups related to cellulose and lignins of the cardoon fraction are also observed [37,43,46].

These findings are in accordance with other studies on polyurethane composites with lignocellulosic materials [35,41,42,43,45,46] and confirm the nature of the produced material.

**Figure 4:**
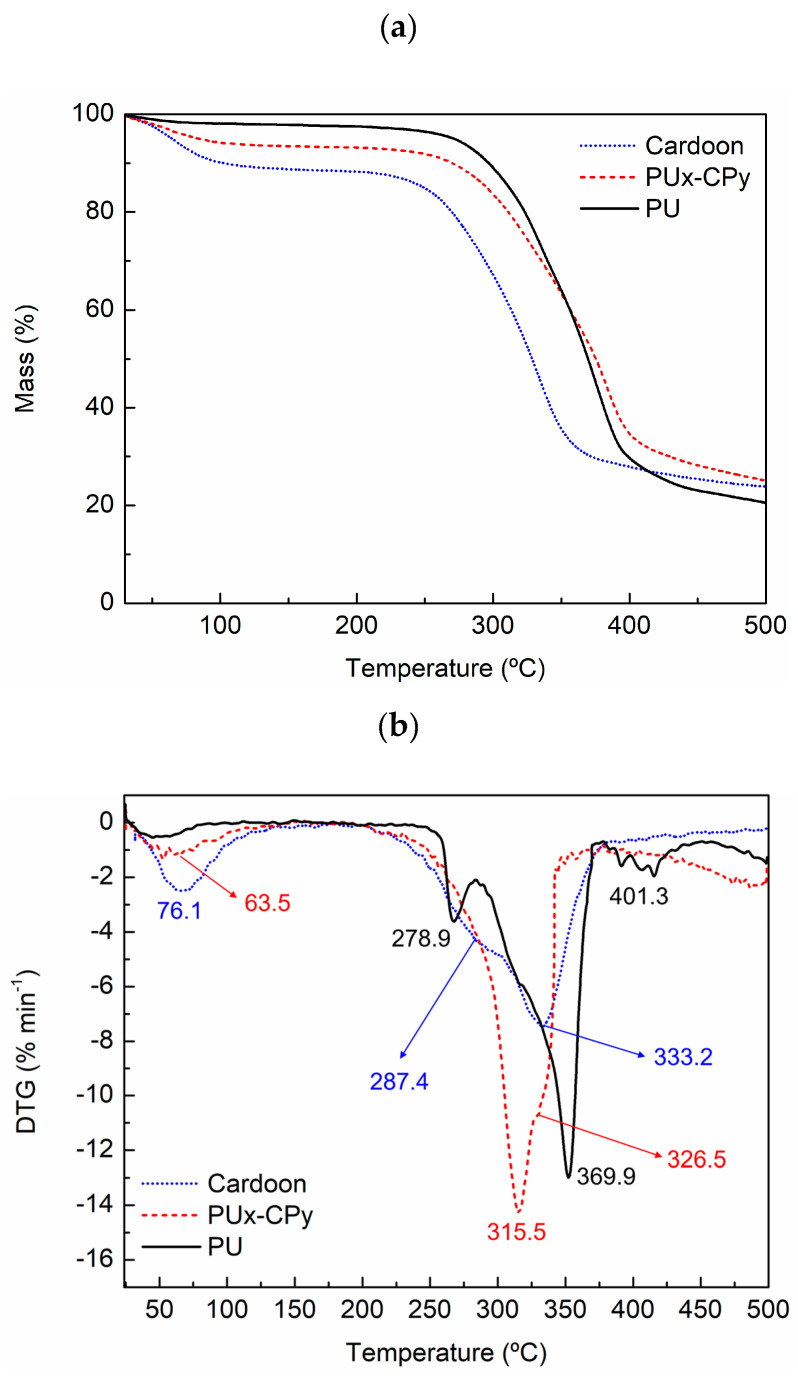
(**a**) TG curve and (**b**) TG first derivative of cardoon (doted blue line), PU foam (solid black line) and PUx-CPy composite material (dashed red line).

The thermogravimetric (TG) analysis was carried out to study the thermal behavior of the cardoon particles, PU foam and PUx-CPy composite material (45 wt% of cardoon particles and 55 wt% of PU), and the results are presented in Figure 4.

Cardoon particles suffer a first stage of mass loss between 50 °C and 110 °C (Figure 4a) due to the evaporation of water [11,35]. In the case of PU foam, this phenomenon has a slight impact, with less than 3% of weight loss (Figure 4b). Moreover, a second loss of weight occurs between 250 °C and 350 °C (Figure 4a), attributed to the degradation of cellulose, hemicellulose and lignin, which represents a major loss of weight (Figure 4b) [11,35]. Regarding PU foam, the degradation occurs between 250 °C and 400 °C (Figure 4a), in three different stages (Figure 4b): (i) from 250 °C to 285 °C, it is related to the degradation of unreacted polyol; (ii) from 285 °C to 370 °C, it is due to the breakage of urethane bonds; and (iii) from 370 °C to 410 °C, it is attributed to soft segments of PU [11,35,47]. The PUx-CPy composite material has a similar profile of weight loss (Figure 4b), with degradation stages at 50 °C–100 °C and at 250 °C–400 °C (Figure 4a).

### 3.2. Optimization of PUx-CPy Formulation

**Figure 5:**
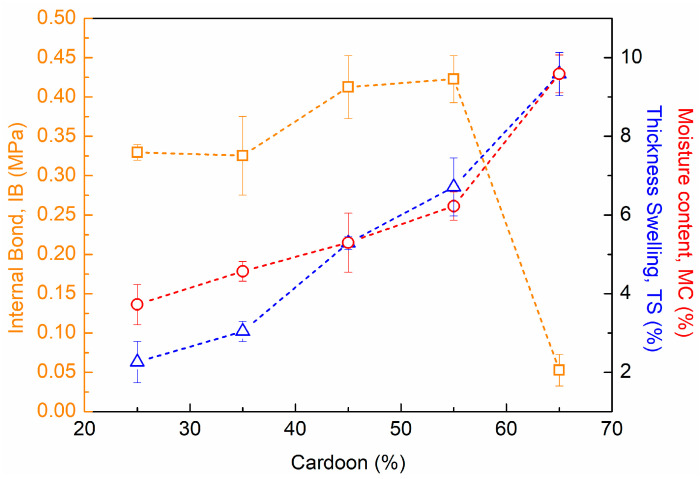
Impact of cardoon content on the physical–mechanical performance of PU75-CPy composite materials: internal bond strength (IB, squares), thickness swelling (TS, triangles) and moisture content (MC, circles).

The physical–mechanical properties of composite materials based on mixtures of resins and lignocellulosic raw materials are highly dependent on the ratio between them. Therefore, it is mandatory to optimize the formulation of the composites to achieve the best performance. In this way, the content of cardoon particles in the formulation of PUx-CPy composite panels was optimized. For this, composite panels with a density of (305 ± 21) kg m^−3^ were produced, using a ratio of 100:75 (*w*/*w*) for polyol and MDI, respectively, and the physical–mechanical performance was evaluated, namely moisture content (MC, %), internal bond strength (IB, MPa) and thickness swelling (TS, %). The obtained results for all PU75-CPy panels are presented in Figure 5.

According to the analysis of Figure 5, the physical–mechanical properties are widely influenced by the content of cardoon particles in the PU75-CPy composite panels. As expected, increasing the fraction of cardoon was reflected in the increase in the MC of composite panels, from 3.7% (PU75-CP25, Figure 5) to 9.6% (PU75-CP65, Figure 5). This finding can be explained by the equilibrium moisture of cardoon particles (around 10%) and the negligible water content in PU foam (<1.5%, see TGA analysis). The TS variation was also in line with this tendency, with a sharp increase from 2.3% (PU75-CP25, Figure 5) to 9.7% (PU75-CP65, Figure 5). As is known, cardoon by-products have a hydrophilic character due to their lignocellulosic nature, which facilitates water absorption [21,41,46,48]. By increasing the cardoon fraction, the ability of composite materials to absorb water also increases (up to saturation point) and, consequently, the TS is negatively affected [21,41,46,48]. The cellulose and hemicellulose fractions of lignocellulosic materials, such as cardoon, are the components most responsible for water absorption and TS due to their high number of hydroxyl groups available to establish hydrogen bonds [46].

Regarding IB variation, the impact of cardoon content is notorious (Figure 5). Up to 55 wt% of cardoon (PU75-CP55), IB was improved c.a. 30%, from 0.33 MPa (PU75-CP25, Figure 5) to 0.43 MPa (PU75-CP55, Figure 5). This effect can be attributed to the reinforcement character of cardoon particles into the PUx-CPy composites [19,30,34,37]. With the increase in the cardoon fraction from 55 wt% to 65 wt%, a sharp decrease was observed in the mechanical properties of the composite material PU75-CP65 (Figure 5). According to the literature, this finding is attributed to the formation of agglomerates of cardoon particles, which reduce the interface of wood/polyurethane and, consequently, limits the load transfer between them. In this situation, the composite material has less resistance to tension forces, and it is more susceptible to cracks and rupture [42,43,45,49].

Considering the impact of cardoon fraction on the physical–mechanical performance of PUx-CPy materials, 45 wt% of cardoon was selected as the optimal condition due to the high IB and low TS.

**Figure 6:**
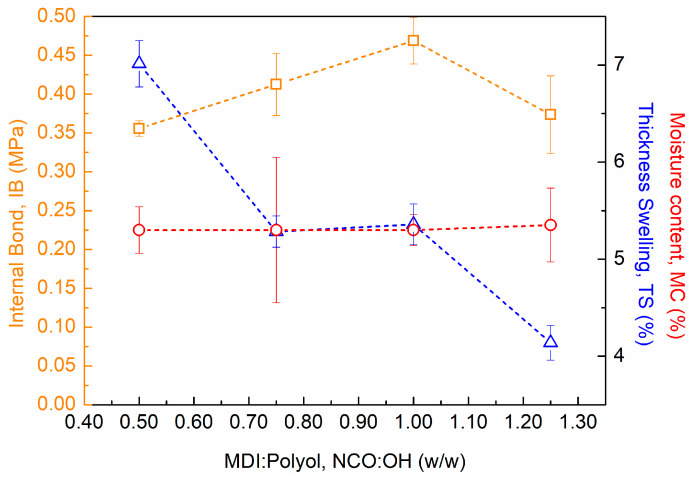
Impact of MDI/polyol ratio on the mechanical performance of PUx-CP45 composite materials: internal bond strength (IB, squares), moisture content (MC, circles), and thickness swelling (TS, triangles).

Taking into account the chemical process used to produce PU foam, additional tests were performed to study the impact of MDI content on the PUx-CPy materials. Therefore, composites with (326 ± 18) kg m^−3^ of density were produced. The formulation adopted was 45 wt% of cardoon and 55 wt% of PU foam, where the MDI content was varied from 50 to 125 wt% (regarding the mass of polyol). The obtained results of MC, TS and IB of all developed materials are presented in Figure 6.

As expected, the MDI content did not affect the MC of composite panels, with all materials reaching c.a. 5.3% (Figure 6). On the other hand, both TS and IB were widely affected by the MDI content. In the case of TS, a noticeable decrease was observed with the increase in MDI (Figure 6). According to the literature, higher amounts of isocyanate derivatives in the polyurethane composition promote urethane bonds through crosslinking reactions between the isocyanate and hydroxyl groups. This specific type of linkages is highly stable, making the produced materials more rigid, and less susceptible to dimensional variations, and consequently, to swelling [35,43,44,45]. A similar behavior was also evidenced in the mechanical performance of composite materials. From 50 to 100% of MDI, the IB value suffered an increment of c.a. 32% (from 0.35 MPa to 0.47 MPa, Figure 6), indicating an improvement in the intermolecular cohesion of the polyurethane matrix caused by the higher crosslinking density [35,43,44,45]. However, with an MDI content of 125%, the mechanical performance of composite material was significantly affected (c.a. 0.38 MPa, Figure 6). A possible explanation is the reduced length between the crosslinking points, which restricts the orientation of additional polymeric chains and decreases the resistance to tension strengths [35,43].

Considering all the results, the PU75-CP45 composite material was selected as the most promising material for the construction industry, as it fulfils the requirements of CEN/TS 16368 for lightweight particleboard types LP1 and LP2. Therefore, further insulation studies were performed.

### 3.3. Insulation Properties

**Figure 7:**
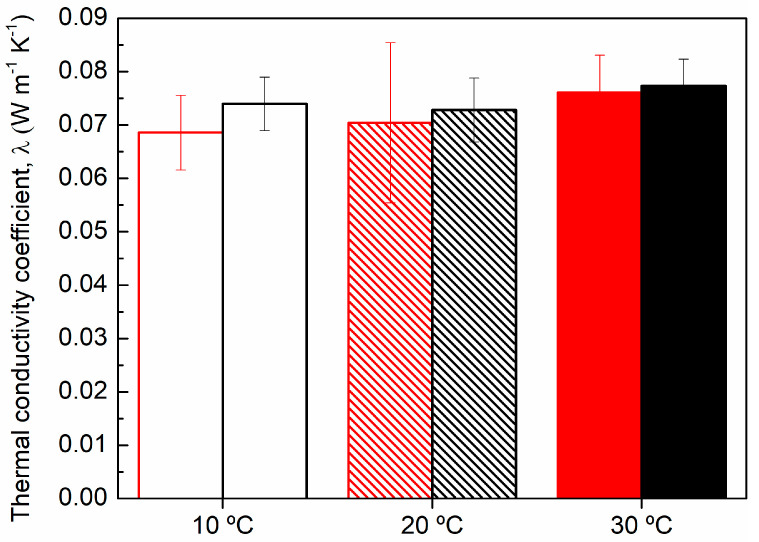
Thermal conductivity of PU75-CP45 composite material at 10 °C (open bars), 20 °C (dashed bars) and 30 °C (solid bars) with 250 (red bars) and 300 kg m^−3^ (black bars) of density.

Thermal conductivity is a key parameter adopted to assess the insulator properties of several types of materials. Therefore, the thermal conductivity coefficient (λ) was determined at 10 °C, 20 °C and 30 °C for the PU75-CP45 composite material. As density has a great impact on insulating properties, an additional sample with lower density (250 kg m^−3^) was also evaluated. The results are presented in Figure 7.

According to conductivity tests, all PU75-CP45 composite materials present λ between 0.068 and 0.077 W m^−2^ K^−1^ (Figure 7). As expected, lowering the density from 300 (black bars, Figure 7) to 250 kg m^−3^ (red bars, Figure 7) favored the insulating behavior of the composite material (lower λ; Figure 7) due to the changes that occurred on its porosity [13,14,42,46,50]. Therefore, PU75-CP45 with 250 kg m^−3^ of density was compared with conventional materials used in the construction industry in terms of thermal insulation capacity (Table 2).

**Figure 8:**
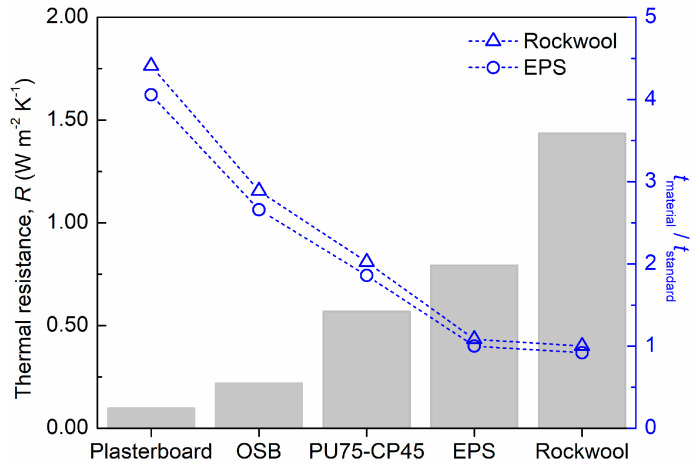
Thermal resistance at 20 °C of PU75-CP45 and other materials (bars). Thickness (e) ratio of all materials, selecting rockwool (triangles) or EPS (circles) as standard material.

As can be observed, the developed composite material has lower thermal conductivity than plasterboard and oriented strand board (OSB) for all studied temperatures (Table 2). However, EPS and rockwool reached much lower values (Table 2). As is known, thickness has a determinant contribution to the thermal conductivity of materials and it should be taken into account when insulation capacity is being compared [51,52]. Therefore, to better compare the insulation capacity of the developed composite material with the conventional solutions, the thermal resistance (R) of all materials was calculated according to Equation (1) [51,52]:

(1) $$R(Wm^{-2}K^{-1})=\frac{t}{\lambda }$$

where λ_20 °C_ is the thermal conductivity coefficient at 20 °C (W m^−1^ K^−1^) and t is the thickness (m) of each material. Moreover, the thickness required to achieve a thermal resistance equivalent to rockwool and EPS was also determined for each material. The obtained results are presented in Figure 8.

The R-values followed the same tendency as λ, with the PU75-CP45 composite material exhibiting a higher insulation capacity than that of plasterboard and OSB, but lower than that of EPS and rockwool (Figure 8). By increasing the thickness of PU75-CP45 from 40 mm to 56 mm and 101 mm (Figure 8), the composite material may present an insulation performance similar to that of EPS and rockwool, respectively. Nevertheless, PU75-CP45 presents important advantages when compared to these materials, namely its wood-based nature and high resistance to deformation. In order to assess the stability properties of PU75-CP45, additional tests of resistance to water and gas emissions (formaldehyde and volatile organic compounds (VOCs)) were also carried out.

### 3.4. Moisture Resistance

The PU75-CP45 composite material (250 kg m^−3^ of density) was evaluated in terms of resistance to water. For this, three different tests were carried out: (i) dimensional stability (30%, 65% and 85% of relative humidity), (ii) the cycle test (immersion, freezing, drying and cooling), and (iii) the boiling test. After the tests, the samples were evaluated in terms of TS, length variation and IB, and the results are presented in Table 3 and Table 4.

As can be observed, the PU75-CP45 composite material exhibited a high dimensional stability at different conditions of relative humidity, with TS and length variation lower than 2% and 4 mm m^−1^, respectively (Table 3). Under tougher conditions (cycle and boiling tests), the composite material also revealed an interesting performance, with 2–4% of TS and c.a. 0.10 MPa of IB (Table 4). Although polystyrene has great resistance to water absorption [53], rockwool suffers tremendous swelling when immersed in water or subjected to higher humidity conditions [6,54]. In this way, the PU75-CP45 composite material may be a valuable alternative for thermal insulation at humid environments. In fact, the values of IB and TS obtained after the boiling and cyclic tests allow us to consider the PU75-CP45 composite material for non-structural uses in humid environments (PB type P3, [22]).

### 3.5. Formaldehyde and Volatile Organic Compounds (VOCs) Emissions

Formaldehyde and volatile organic compounds (VOCs) have been recognized as contaminants of indoor air since the 20th century [55,56]. Formaldehyde is known to have carcinogenic and mutagenic properties and can act as a toxicant and skin sensitizer. The major sources of these compounds in the interior of buildings are construction materials, such as particleboards, plywood, flooring, laminate, among others [55,56]. In fact, the harmful effects of formaldehyde and VOCs in the environment and human health are so serious that the European Commission has established limits for their emission (European Commission regulation 2023/1464 of 14 July 2023). The new rules establish an emission limit of 0.062 mg m^−3^ of formaldehyde into indoor air for the largest contributors, such as wood-based articles and furniture. Therefore, PU75-CP45 was evaluated in terms of formaldehyde using the gas analysis method [31] and VOCs [32] emissions, and the results are presented in Table 5.

As can be observed, the PU75-CP45 composite material presents low emissions of formaldehyde and VOCs (Table 5). In the first case, the developed material reached ten times lower emissions of formaldehyde (0.37 mg m^−2^ h^−1^; Table 5) than the maximum limit defined for E1 class ([57], Table 5). In the case of VOCs emissions, the value was lower than 5.0 µg of toluene eq. m^−3^, being classified as a very-low-emission material (EC1PLUS class according EMICODE^®^ [58], Table 5). These results demonstrate that the PU75-CP45 composite material has no significant danger to human health and to the environment, making it a safe alternative for use in the construction industry.

### 3.6. Susceptibility to Biological Degradation

In order to assess the susceptibility of the PU75-CP45 composite material to biological deterioration, the samples were subjected to tests with subterranean termites (*R. grassei*) and white rot fungi (*P. sanguineus*). In Table 6 and Table 7, the results (average values) of both tests are presented, respectively.

As can be observed, the PU75-CP45 composite material presented a moisture content c.a. 22% (Table 6) similar to that of the control material. However, the number of termites that survive after 4 weeks of contact with the PU75-CP45 composite material was considerably lower (c.a. 20%, Table 6) when compared to untreated *F. sylvatica* L. wood. On the other hand, the mass loss observed was significantly higher in the control samples (c.a. 25%, Table 6) by comparing with that of the PU75-CP45 composite material (c.a. 7%, Table 6). Regarding susceptibility to white rot fungi (*P. sanguineus*), the same tendency was observed, with the PU75-CP45 composite material exhibiting lower mass loss than *F. sylvatica* L. (Table 7).

These results might be explained by the formaldehyde and VOCs emission of the composite material (see Section 3.5) that can be hazardous to macro- and micro-organisms (*R. grassei* and *P. sanguineus*) and decrease their survival.

## 4. Conclusions

A composite material with c.a. 300 kg m^−3^ of density was produced by mixing cardoon particles with polyurethane (PU) adhesive. The presence of both components was morphologically and chemically confirmed through SEM and FTIR, respectively. The foaming of polyurethane enabled the creation of a low-density material, with good cohesion and resistance. The evaluation of internal bond strength (IB) and thickness swelling (TS) allowed for the optimization of the content of cardoon in the formulation of the composite and the ratio between isocyanate and polyol. An increasing cardoon fraction decreases the resistance of the composite material to water (higher TS). Contrariwise, a higher amount of polyol in the PU formulation reduces TS. Both parameters allowed for the tuning of the mechanical performance of the composite material. The best-performing composite was PU75-CP45, with 45% wt of cardoon particles and 75% of isocyanate, achieving an IB of 0.41 MPa and a TS of 5.3%. By decreasing the density from 300 to 250 kg m^−3^, the thermal insulation properties were enhanced, reaching c.a. 0.070 W m^−1^ K^−1^ and 0.568 W m^−2^ K^−1^ of thermal conduction and resistance, respectively. When compared to other conventional materials used in the construction industry, the PU75-CP45 composite exhibits better insulation capacity than plasterboard and OSB. With the increase in its thickness, the composite may achieve insulation performances near those of rockwool and EPS. Regarding the impact on indoor air quality, the PU75-CP45 composite material has a very low emission of formaldehyde and VOCs, which is determinant for its use in the construction industry. Additionally, the PU75-CP45 composite material has a lower tendency to suffer biological degradation than that of untreated wood.

## Figures and Tables

**Table 1:** Formulation (cardoon, polyol and MDI mass and isocyanate index) of each composite panels PUx_CPy.

Composite	Cardoon Mass (g)	Polyol Mass (g)	MDI Mass (g)	Isocyanate Index
PU75_CP25	2062.5	1221.4	916.1	105
PU75_CP35	2887.5	1221.4	916.1	105
PU75_CP45	3712.5	1221.4	916.1	105
PU75_CP55	4537.5	1221.4	916.1	105
PU75_CP65	5362.5	1221.4	916.1	105
PU50_CP45	3712.5	2525.0	1262.5	70
PU100_CP45	3712.5	1893.8	1893.8	140
PU125_CP45	3712.5	1683.3	2104.2	175

**Table 2:** Thickness (t) and thermal conductivity coefficient (λ) at 10 °C, 20 °C and 30 °C experimentally determined for PU75-CP45 composite material (250 kg m^−3^) and different materials used in the construction industry.

Material	t (mm)	λ_10 °C_ (W m^−1^ K^−1^)	λ_20 °C_ (W m^−1^ K^−1^)	λ_30 °C_ (W m^−1^ K^−1^)
Plasterboard	15	0.15284	0.15349	0.15441
OSB	22	0.0984	0.1006	0.10295
PU75-CP45	40	0.06858	0.07041	0.07608
Expanded polystyrene (EPS)	30	0.03642	0.03784	0.03924
Rockwool	50	0.03357	0.03483	0.03636

**Table 3:** Thickness swelling (TS) and length variation in PU75-CP45 composite material in the evaluation of dimensional stability.

Conditions	Change in Thickness (%)	Change in Length (mm m^−1^)
From 65 to 30% of RH	−0.7 ± 0.3	−2.7 ± 0.3
From 65 to 85% of RH	1.1 ± 0.1	3.1 ± 0.2

**Table 4:** Thickness swelling (TS) and internal bond (IB) strength of PU75-CP45 composite material before and after cycle and boiling tests.

Test	Thickness Swelling, TS (%)	Internal Bond Strength, IB (MPa)
Cyclic test	2.4 ± 0.1	0.10 ± 0.03
Boiling test	3.6 ± 0.2	0.10 ± 0.02

**Table 5:** Formaldehyde and VOCs emissions of PU75-CP45 composite material and its classification according to the standard requirements.

Test	Value	Classification
Formaldehyde emission	(0.37 ± 0.01) mg m^−2^ h^−1^	E1 (1)
VOCs emission	<5.0 µg of toluene eq. m^−3^	EC1PLUS (2)

(1) According to EN 13986:2004+A1:2015 [57] (<3.5 mg m^−2^ h^−1^); (2) according to EMICODE^®^ (<60 µg m^−3^ after 28 days).

**Table 6:** Susceptibility of PU75-CP45 composite material to *R. grassei* (*n* = 3): moisture content, survival, mass loss and grade of attack. *Fagus sylvatica* L. was used as control.

Material	Moisture Content (%)	Survival (%)	Mass Loss (%)	Grade of Attack
PU75-CP45	21.5 ± 13.1	19.9 ± 23.6	6.5 ± 4.0	4
*Fagus sylvatica* L.	26.3 ± 17.7	72.9 ± 17.5	24.8 ± 3.7	4

**Table 7:** Susceptibility of PU75-CP45 composite material to white rot fungi *P. sanguineus* (n = 10): moisture content and mass loss. *Fagus sylvatica* L. was used as control.

Material	Moisture Content (%)	Mass Loss (%)
PU75-CP45	114.2 ± 24.8	11.9 ± 1.5
*Fagus sylvatica* L.	78.2 ± 47.0	41.3 ± 8.3

## Data Availability

Data are contained within the article.

## References

[1] Sandanayake M.S. (2022). Environmental Impacts of Construction in Building Industry—A Review of Knowledge Advances, Gaps and Future Directions. Knowledge.

[2] Dräger P., Letmathe P. (2022). Value losses and environmental impacts in the construction industry—Tradeoffs or correlates?. J. Clean. Prod..

[3] Anh L.D.H., Pásztory Z. (2021). An overview of factors influencing thermal conductivity of building insulation materials. J. Build. Eng..

[4] European Commission (2021). Key Proposals for Homes and Buildings: Making Our Homes and Buildings Fit for a Greener Future.

[5] Ulutaş A., Balo F., Topal A. (2023). Identifying the Most Efficient Natural Fibre for Common Commercial Building Insulation Materials with an Integrated PSI, MEREC, LOPCOW and MCRAT Model. Polymers.

[6] Ducoulombier L., Lafhaj Z. (2017). Comparative study of hygrothermal properties of five thermal insulation materials. Case Stud. Therm. Eng..

[7] Abu-Jdayil B., Mourad A.H., Hittini W., Hassan M., Hameedi S. (2019). Traditional, state-of-the-art and renewable thermal building insulation materials: An overview. Constr. Build. Mater..

[8] Aditya L., Mahlia T.M.I., Rismanchi B., Ng H.M., Hasan M.H., Metselaar H.S.C., Muraza O., Aditiya H.B. (2017). A review on insulation materials for energy conservation in buildings. Renew. Sustain. Energy Rev..

[9] Florea I., Manea D.L. (2019). Analysis of Thermal Insulation Building Materials Based on Natural Fibers. Procedia Manuf..

[10] Bozsaky D. (2019). Nature-Based Thermal Insulation Materials from Renewable Resources—A State-Of-The-Art Review. Slovak J. Civ. Eng..

[11] Bellel N., Bellel N. (2023). Sustainable heat insulation composites based on Portland cement reinforced with date palm fibers. J. Eng. Fiber. Fabr..

[12] Belhous M., Boumhaout M., Oukach S., Hamdi H. (2023). Effect of a Material Based on Date Palm Fibers on the Thermal Behavior of a Residential Building in the Atlantic Climate of Morocco. Sustainability.

[13] Bakatovich A., Gaspar F. (2019). Composite material for thermal insulation based on moss raw material. Constr. Build. Mater..

[14] Hussain A., Calabria-Holley J., Lawrence M., Jiang Y. (2019). Hygrothermal and mechanical characterisation of novel hemp shiv based thermal insulation composites. Constr. Build. Mater..

[15] Ahmad M.R., Chen B., Haque M.A., Ali Shah S.F. (2019). Development of a sustainable and innovant hygrothermal bio-composite featuring the enhanced mechanical properties. J. Clean. Prod..

[16] Parlato M.C.M., Valenti F., Lanza E., Porto S.M.C. (2022). Spatial analysis to quantify and localise the residual cardoon stem fibres as potential bio-reinforcements for building materials. Int. J. Sustain. Eng..

[17] Barracosa P., Barracosa M., Pires E. (2019). Cardoon as a Sustainable Crop for Biomass and Bioactive Compounds Production. Chem. Biodivers..

[18] Santos J., Pereira J., Ferra J., Magalhães F.D., Martins J.M., Carvalho L.H. (2021). New Cardoon (*Cynara cardunculus* L.) Particleboards Using Cardoon Leaf Extract and Citric Acid as Bio-adhesive. Mater. Circ. Econ..

[19] Barbosa C.H., Andrade M.A., Vilarinho F., Castanheira I., Fernando A.L., Loizzo M.R., Silva A.S. (2020). A new insight on cardoon: Exploring new uses besides cheese making with a view to zero waste. Foods.

[20] Monteiro S., Nunes L., Martins J., Magalhães F.D., Carvalho L. (2020). Low-density cardoon (*Cynara cardunculus* L.) particleboards bound with potato starch-based adhesive. Polymers.

[21] Monteiro S., Martins J., Magalhães F.D., Carvalho L. (2016). Low density wood-based particleboards bonded with foamable sour cassava starch: Preliminary studies. Polymers.

[22] (2014). Lightweight Particleboards-Specifications.

[23] (1993). Wood-Based Panels–Determination of Density.

[24] (1993). Wood-Based Panels-Determination of Moisture Content.

[25] (1993). Particleboards and Fibreboards-Determination of Tensile Strength Perpendicular to the Plane of the Board.

[26] (1993). Particleboards and Fibreboards-Determination of Swelling in Thickness after Immersion in Water.

[27] (1993). Fibreboards-Determination of Dimensional Changes Associated with Changes in Relative Humidity.

[28] (1993). Fibreboards. Cyclic Tests in Humid Conditions.

[29] (1995). Particleboards-Determination of Moisture Resistance-Part 1: Boil Test.

[30] (2001). Thermal Performance of Building Materials and Products-Determination of Thermal Resistance by Means of Guarded Hot Plate and Heat Flow Meter Methods-Products of High and Medium Thermal Resistance.

[31] (2023). Wood-Based Panels-Determination of Formaldehyde Release-Part 3: Gas Analysis Method.

[32] (2006). Indoor Air-Part 9: Determination of the Emission of Volatile Organic Compounds from Building Products and Furnishing Emission Test Chamber Method.

[33] (2012). Wood Preservatives-Determination of Toxic Values Against Reticulitermes Species (European Termites) (Laboratory Method).

[34] Verma P., Dyckmans J., Militz H., Mai C. (2008). Determination of fungal activity in modified wood by means of micro-calorimetry and determination of total esterase activity. Appl. Microbiol. Biotechnol..

[35] Olszewski A., Kosmela P., Piszczyk Ł. (2022). Synthesis and characterization of biopolyols through biomass liquefaction of wood shavings and their application in the preparation of polyurethane wood composites. Eur. J. Wood Wood Prod..

[36] De Souza F.M., Kahol P.K., Gupta R.K. (2021). Introduction to Polyurethane Chemistry. Polyurethane Chem. Renew. Polyols Isocyanates.

[37] Reignier J., Alcouffe P., Méchin F., Fenouillot F. (2019). The morphology of rigid polyurethane foam matrix and its evolution with time during foaming—New insight by cryogenic scanning electron microscopy. J. Colloid Interface Sci..

[38] Sharmin E., Zafar F. (2012). Polyurethane: An Introduction. Polyurethane.

[39] Crescentini T.M., May J.C., McLean J.A., Hercules D.M. (2019). Mass spectrometry of polyurethanes. Polymer.

[40] Pongmuksuwan P., Harnnarongchai W. (2022). Synthesis and characterization of soft polyurethane for pressure ulcer prevention. Polym. Test..

[41] Masturi W.N., Jannah R.M., Maulana T., Darsono T., Sunarno S. (2020). Rustad, Mechanical and physical properties of teak leaves waste/polyurethane composites for particleboard application. Adv. Compos. Lett..

[42] Członka S., Strąkowska A., Kairytė A. (2020). Effect of walnut shells and silanized walnut shells on the mechanical and thermal properties of rigid polyurethane foams. Polym. Test..

[43] Chen Y.C., Tai W. (2018). Castor oil-based polyurethane resin for low-density composites with bamboo charcoal. Polymers.

[44] Džunuzović J.V., Pergal M.V., Jovanović S., Vodnik V.V. (2011). Synthesis and swelling behavior of polyurethane networks based onhyperbranched polymer. Hem. Ind..

[45] Fornasieri M., Alves J.W., Muniz E.C., Ruvolo-Filho A., Otaguro H., Rubira A.F., De Carvalho G.M. (2011). Synthesis and characterization of polyurethane composites of wood waste and polyols from chemically recycled pet. Compos. Part A Appl. Sci. Manuf..

[46] Radzi A.M., Sapuan S.M., Jawaid M., Mansor M.R. (2019). Water absorption, thickness swelling and thermal properties of roselle/sugar palm fibre reinforced thermoplastic polyurethane hybrid composites. J. Mater. Res. Technol..

[47] Zhao S., Pang H., Li Z., Wang Z., Kang H., Zhang W., Zhang S., Li J., Li L. (2021). Polyurethane as high-functionality crosslinker for constructing thermally driven dual-crosslinking plant protein adhesion system with integrated strength and ductility. Chem. Eng. J..

[48] Bartczak P., Stachowiak J., Szmitko M., Grząbka-Zasadzińska A., Borysiak S. (2023). Multifunctional Polyurethane Composites with Coffee Grounds and Wood Sawdust. Materials.

[49] Ciecierska E., Jurczyk-Kowalska M., Bazarnik P., Gloc M., Kulesza M., Kowalski M., Krauze S., Lewandowska M. (2016). Flammability, mechanical properties and structure of rigid polyurethane foams with different types of carbon reinforcing materials. Compos. Struct..

[50] Ghosh S.K., Bairagi S., Kumar Ghosh S., Bhattacharyya R., Mohan Mondal M. (2016). Study on Potential Application of Natural Fibre Made Fabrics as Thermal Insulation Medium. Am. Int. J. Res. Sci..

[51] Choi H.-J., Ahn H., Choi G.-S., Kang J.-S., Huh J.-H. (2021). Analysis of Long-Term Change in the Thermal Resistance of Extruded Insulation Materials through Accelerated Tests. Appl. Sci..

[52] Bae M., Ahn H., Kang J., Choi G., Choi H. (2022). Determination of the Long-Term Thermal Performance of Foam Insulation Materials through Heat and Slicing Acceleration. Polymers.

[53] Cai S., Zhang B., Cremaschi L. (2018). Moisture behavior of polystyrene insulation in below-grade application. Energy Build..

[54] Wu X., Jiang D., Zhang H., Wang K., Ye X., Chen Z., Zhang J. (2020). Study on the Properties of Rock Wool for External Thermal Insulation of Buildings under the Soaking and Hot & Humid. J. Phys. Conf. Ser..

[55] Huang S., Song S., Nielsen C.P., Zhang Y., Xiong J., Weschler L.B., Xie S., Li J. (2022). Residential building materials: An important source of ambient formaldehyde in mainland China. Environ. Int..

[56] Wi S., Kim M.G., Myung S.W., Baik Y.K., Lee K.B., Song H.S., Kwak M.J., Kim S. (2020). Evaluation and analysis of volatile organic compounds and formaldehyde emission of building products in accordance with legal standards: A statistical experimental study. J. Hazard. Mater..

[57] (2015). Wood-Based Panels for Use in Construction-Characteristics, Evaluation of Conformity and Marking.

[58] EMICODE^®^ Classification. https://www.emicode.com/en/emission-classes/.

